# Chronic unpredictable mild stress combined with a high-fat diets aggravates atherosclerosis in rats

**DOI:** 10.1186/1476-511X-13-77

**Published:** 2014-05-10

**Authors:** Shuling Wang, Gao Xiaoling, Li Pingting, Liu Shuqiang, Zeng Yuaner

**Affiliations:** 1 School of Chinese Herb Medicine, Guangzhou University of Chinese Medicine, Guangzhou Higher Education Mega Center, 232 Waihuandong Road, Guangzhou 510006, China

**Keywords:** Chronic unpredictable mild stress, High-fat diet, Reverse cholesterol transport, Atherosclerosis

## Abstract

**Background:**

Depression and high-fat diet are both known as independent risk factors for atherosclerosis and other cardiovascular diseases, suggesting the interaction of psychological and physiological factors in the development of these diseases. The liver is a crucial organ that facilitate lipid metabolism especially in reverse cholesterol transport (RCT), while according to the theory of Traditional Chinese Medicine, depression as a kind of psychological stress has an influence on hepatic function. So there seem to be some links between depression and lipid metabolic disorders.

**Methods:**

To investigate these links, we separately treated rats with chronic unpredictable mild stress (CMS) and/or a high-fat diet (HD) to evaluate the development of atherosclerosis and the expression of hepatic ABCG8, ABCG5, SR-BI, CYP7A1, LXRα, and LCAT which were associated with reverse cholesterol transport.

**Results:**

This study provided evidence that high-fat diet greatly decreased these genes expression related to RCT while chronic stress alone tended to promote RCT. Chronic unpredictable mild stress combined with a high-fat diet attenuated RCT and aggravated atherogenesis.

**Conclusions:**

These observations suggested that chronic psychological stress alone is virtually propitious to lipid metabolism, however when under a condition of high-fat diet, it deteriorated atherosclerotic plague and did harm to RCT.

## Introduction

In modern societies, obesity and regular consumption of high-fat diets is usually associated with the occurrence of metabolic syndrome. Hyperlipidemia is a major underlying reason for the development of cardiovascular diseases [1]. However, high-fat diet alone cannot account for the current epidemic of atherosclerosis and coronary heart disease [2]. Despite the association between atherosclerosis and increase in serum lipid concentration, many individuals develop severe atherosclerotic lesions while they have low serum lipid concentration, and others develop far more severe atherosclerotic lesions than would be expected on the basis of a modest elevation of serum lipids [3].

Growing evidence demonstrates that psychological risk variables can contribute to physical disease [4,5]. Several epidemiologic studies have shown depression to be an independent risk factor for the development of vascular disease in patients, increase morbidity and mortality in patients with preexisting coronary artery disease [6,7]. Some clinical studies have suggested a delayed effect of depression on the vascular system [8]. Pratt and colleagues found that among patients free of heart disease, those who had previously experienced an episode of depression were four times more likely to suffer from cardiac events within the following 14 years [9]. In addition, the risk of myocardial infarction increased up to 10 years after the onset of the first depressive episode [10]. As exposure to chronic stress also correlates with an increasing incidence of visceral obesity, insulin resistance, hypertension and atherosclerosis, stress has been recognized as a risk factor for cardiovascular and metabolic diseases [11].

There is no compelling evidence revealing that chronic psychological stress and negative mood state correlate bidirectionally with metabolic syndrome. In response to stress, some people lose weight while others gain weight. Previous studies have provided conflicting evidences for the association between metabolic syndrome and psychological problems [12]. For instance, it was shown that metabolic syndrome was associated with long-term depressive symptoms, while another study reported absence of any correlation between metabolic syndrome, depression and anxiety. The mechanisms involved in the relationships among chronic stress, visceral obesity, hyperlipidemia and cardiovascular diseases have not yet been completely clarified. According to the theory of Traditional Chinese Medicine, irregular psychological stressors damage physiological functions of liver [13,14]. As it’s known that, liver plays a critical role in reverse cholesterol transport (RCT), which is a key step in atherogenesis and hyperlipidemia. So we hypothesize there would be some links between psychological stress and lipid metabolism, and depressive disorders may exert effects on RCT.

In an effort to thoroughly verify this hypothesis, the present study aims to investigate the influence of depressive disorders, with or without a high-fat diet, on the development of atherosclerosis and expression of relevant hepatic genes associated with RCT in rats. Since rats generally adapt to repeated application of stressors, we applied CMS as a stimulating factor in this study. The chronic unpredictable mild stress (CMS) animal model is valid, reliable and sensitive for studying depressive disorders in rats [15]. This procedure replicates several depression-related behavioral and physiological impairments, which are reversed by antidepressant treatment [16]. We also explored the relationship between chronic stress and serum lipid levels, liver function and pathology.

## Methods

### Animals and chow

Adult male Sprague Dawley rats weighing 180-200 g were obtained from Experimental Animal Center, Guangzhou University of Chinese Medicine (SCXK (Guangdong) 2008–0020). The rats were housed in groups of 4 at their arrival and allowed to become used to their new environment for 1 week. All rats had free access to water and food in standard laboratory conditions (temperature of 25 ± 1°C; 12-hour light/dark cycle, light on at 08:00). Protocols were conducted in accordance with standard ethical guideline, which was approved by the institutional animal ethics committee. Diets were purchased from Beijing Keao third-Feed Co.. Regular chow (ND, Normal diet) is composed of 4% saturated, 4.6% mono-unsaturated and 2.4% polyunsaturated fat, and 45% carbohydrates, totalizing 11.7% of kcal from fat. High fat diet (HD) is composed of 90.45% basic feed and 2% cholesterol, 0.35% cholate, 0.2% propylthiouracil, 5% lard and 2% sugar.

### General procedure

The rats were randomized into the control group, high-fat group (HD), CMS group and CMS + HD group (n = 10 in each group). Rats in the control group and CMS group were fed a standard diet, formulated for the maintenance of adult rodents. The HD and CMS + HD group received a modified high-fat but not high-sugar diet, based on a purified moderately kept in standard laboratory conditions. In the present study, 8-week CMS procedure without any antidepressant treatment was used. Rats in control group and HD group were housed in groups of 4 in standard laboratory cages (42 cm × 28 cm × 18 cm) and fed with a high-fat diet, while rats in CMS group and CMS + HD group were singularly housed in individual cages (8 cm × 13.5 cm × 8.1 cm). The body weight of each animal was evaluated every Monday in a blind manner. During the last week, after the open-field test, the rats were euthanized for a biochemical analysis and vascular reactivity study.

### Chronic unpredictable mild stress (CMS)

The CMS regimen used is a variation of the procedure previously described [17] with a slight modification. The control group rats were housed together without disturbance, except for application of the necessary procedures, while rats of other groups were singularly housed and exposed to the following stressors in a random order every day between 9:30 and 12:00 a.m. for 8 weeks: cage tilting (45˚),damp sawdust for 24 hours (200 ml of water per individual cage, which is enough to make the sawdust bedding wet), noises for 1 hour (alternative periods of 60 dBA noise for 10 minutes and 10 minutes of silence), swimming in 4°C cold water for 5 minutes, exposure to an experimental room at 50°C for 5 minutes, 24 hours of food deprivation and 24 hours of water deprivation, respectively, tail clamp for 1 minute, 15 unpredictable shocks (15 MA, one shock/5 s, 10 s duration) and restricted movement for 4 hours.

### Assessment of depressive-like behavior

#### Food intake and sucrose preference

Food intake was assessed on the day before the end of experimental procedure, and measuring results were corrected by rats’ body weight. The *sucrose preference* test was performed as previously described [18]. At the beginning of the test all the groups were singly housed during 48 h in individual cages (see housing Conditions). Two bottles were available in each cage, one with 200 ml of 32% sucrose (w/v) and the other also with 200 ml of tap water. Preference was measured as follows: (1) sucrose consumption (ml) 200 ml; (2) water consumption (ml) 200 ml; and (3) total liquid, sucrose consumption + water consumption. At the end of 48th hour, the bottles were removed, the consumption was noted and the animals were returned to their previous housing conditions.

#### Open-field test

Open-field test was conducted as described by Dunn [19], to assess chronic stress effects on rats. The test established to assess CMS-induced effects on animals’ motivational behavior, was performed on the last 2 days before the end of the experiment. The apparatus consists of a rectangular box (80 cm × 80 cm × 50 cm), a floor divided into 25 (16 cm × 16 cm) identical squares. The rats were placed in the center of the open-field and allowed to freely explore for 3 minutes. Two motor parameters were quantified throughout this test: locomotion frequency (numbers of with which the rat crossed one of the grid lines with all four paws) and rearing frequency (times of rearing with hind legs). The animals were acclimated to the experimental room for at least 2 hours prior to the beginning of the open-field test. Open-field test was carried out in a soundproof room without any human interference and cleaned with a 5% water-ethanol solution before behavioral testing to eliminate bias due to odors left by previous rats. The evaluation of rat’s motivational behavior was performed in a blinded manner by two independent observers. After testing inter-observer reliability, the mean from the results was statistically analyzed.

### Biochemical analysis

Blood samples were collected under chloral hydrate anesthesia and centrifuged at 3500 rpm for 15 minutes by a refrigerated centrifuge (HEMA, China), and the serum was transferred into a separate vial and stored at 4°C. Serum concentrations of total cholesterol (TC), low-density lipoprotein cholesterol (LDL-C), high-density lipoprotein cholesterol (HDL-C), albumin (ALB), globulin (GLB) and alkaline phosphatase (ALP) were measured by enzymatic assays using an automated biochemical analyzer (Cobas 8000, Roche, Germany).

### Histology of abdominal aorta and liver

After blood collection liver tissue was immediately removed and weighed, while abdominal aorta was dissected and cut into parts. A part of liver was snap-frozen in liquid nitrogen and stored an −80°C for RNA extraction and Western blot analysis; the remaining part and aorta were fixed in a 4% poly-formaldehyde solution and processed for paraffin embedding and sectioning. After fixed in a 4% poly-formaldehyde solution embedded in paraffin wax, aorta and liver were cut into 5-μm-thick transverse sections and stained with hematoxylin-eosin (HE). The measurements of intima thickness, media thickness, total intima-media thickness (IMT), and wall thickness were made in HE stained sections of abdominal aorta under a microscope (400 × magnifications; SMART, Chongqing, China) using Image Pro Plus 6.0 software.

### Quantitative real-time PCR

Total RNA was extracted with Trizol reagent (Invitrogen, Shanghai, China). The quality of samples was confirmed by agarose gel electrophoresis. Total RNA was reverse transcribed to complementary DNA using Prime Script® 1st Strand cDNA Synthesis Kit (TAKARA, Kyoto, Japan). The thermo cycler settings were 30°C for 10 minutes, 42°C for 60 minutes, and 70°C for 15 minutes. Real-time PCR was performed in triplicates using SYBR® Premix Ex Taq kit (TAKARA) in a 25 μL reaction volume. GAPDH was used as the housekeeping gene. The primers used for real-time PCR were listed in Table 1. Relative mRNA levels were calculated by the method of 2^-∆∆Ct^.

**Table 1:** Primers used for real-time PCR analysis

**Gene**	**Primer**	**Sequence (5′→3′)**
LCAT	Sense	TGTAGCCACACGTAGCACTG
	Anti-sense	AGGCACCCAGTAGGATAGCA
CYP7A1	Sense	GGAAAAGCTGGCTGAGGGAT
	Anti-sense	TCAAAGGTGGAGAGCGTGTC
LXR_α_	Sense	TACAATGTGCTGAGCTGCGA
	Anti-sense	TGTAGCGGTCTCCCTTGATG
SR-B1	Sense	CCCCATGAACTGTTCCGTGA
	Anti-sense	CCACAGCAATGGCAGGACTA
ABCG5	Sense	GGGAAGTGTTTGTGAACGGC
	Anti-sense	GTGTATCTCAGCGTCTCCCG
ABCG8	Sense	ACGTGGACTTGACGAGCATT
	Anti-sense	GTGTCCTGTGTGAGGGTCTG
GAPDH	Sense	TGCTGGGGCTGGCATTGCTCTC
	Anti-sense	ATGAGGTCCACCACCCTGTTGC

### Western blot analysis

Liver tissues were frozen in liquid nitrogen. Total protein was isolated in a lysis buffer, resolved by 10% sodium dodecyl sulfate polyacrylamide gel electrophoresis (SDS-PAGE) and transferred onto polyvinylidene fluoride (PVDF) membranes by electroblotting. LCAT, CYP7A1, LXR_α,_ SR-BI, ABCG5 and ABCG8 proteins were detected using an anti-LCAT polyclonal antibody (1:1500 dilution, Proteintech, Wuhan), anti-CYP7A1 polyclonal antibody (1:500 dilution, Bioss, Beijing, China), anti- LXR_α_ polyclonal antibody (1:300 dilution, Proteintech, Wuhan, China), anti- SR-BImonoclonal antibody (1:500 dilution, Abcam, UK), anti-ABCG5 polyclonal antibody (1:500 dilution, Bioss, Beijing, China) and anti-ABCG8 polyclonal antibody (1:500 dilution, Bioss, Beijing, China). The bands were visualized with an enhanced chemiluminescence kit (Millipore, Billerica, MA) and analyzed with Image-Pro Plus 6.0 (Media Cybernetics, US).

### Statistical analysis

The results are presented as mean ± SEM. Statistical significance was determined by t-Student’s test when two groups were compared or by ANOVA and post-hoc two-tailed Newman-Keuls test when more than two groups were compared. A *p* value of less than 0.05 was considered statistically significant.

## Results

### CMS exposure resulted in depression-like symptoms both in normal diet and in high-fat diet rats

**Figure 1:**
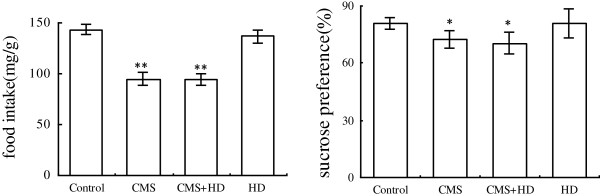
**Effects of chronic psychological stress and high-fat diet on food intake and sucrose preference in rats.** Food intake and sucrose preference were determined two days before the end of experimental procedure. Data were mean ± SEM. ^*^P < 0.05, ^**^P < 0.001 vs. control group, one–way ANOVA (n = 10 per group/time point).

**Figure 2:**
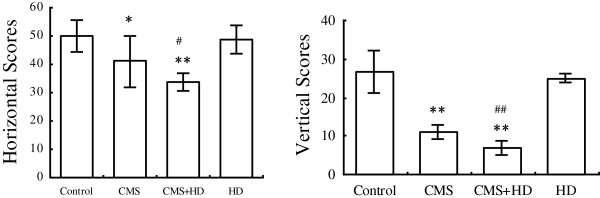
**Comparison of behavioral motor effects elicited by chronic unpredictable mild stress, high-fat diet or both in open-field test.** The data were obtained one day before rats were sacrificed. Values are expressed as the mean ± SEM (n = 10/group). ^*^P < 0.05 and ^**^P < 0.001 compared to the control group. ^#^P < 0.05 and ^##^P < 0.01 compared to CMS group. One-way ANOVA followed by Newman-Keuls test.

At beginning of the experiment, all rats in each group essentially had the same condition. During the experimental period of 8 weeks, ANOVA revealed a significant main effect of CMS in depression-like behavior test. The CMS protocol resulted in a significant deterioration of food intake and sucrose preference compared with control and HD groups. There was no marketable difference between CMS group and CMS + HD group in these tests (Figure 1). To assess whether the CMS procedure with/without a high-fat diet could cause changes in exploratory or locomotor behavior of rats, the rats were exposed to an open-field test. Stressed rats from the CMS and CMS + HFD groups exhibited significantly less horizontal and vertical scores compared with controls (P < 0.05, P < 0.01), indicated that their locomotor and exploratory activity were weaker than controls. However, treatment with high-fat diet alone did not cause any alteration in this behavior, and rats in CMS + HD group got much less scores both in horizontal (P < 0.05) and vertical motion (P < 0.01) than in CMS group (Figure 2).

### Effects of high fat diet and chronic stress on lipid metabolism

**Figure 3:**
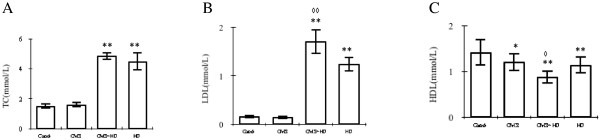
**Effects of stress on plasma lipid concentrations of TC (A), LDL (B) and HDL(C) in control rats (control), stressed rats (CMS), non-stressed rats fed with HD (HD), and stressed rats fed with HD (CMS + HD)(n = 10 rats per group).** Data were mean ± SEM. ^*^P < 0.05, ^**^P < 0.01: compared to control group; ^◊^*P* < 0.05, ^◊◊^*P* < 0.01: compared to HD group (one-way ANOVA).

The level of TC and LDL in serum of both HD and CMS + HD group is remarkably higher than control group (P < 0.01), while HDL is much lower (P < 0.01). CMS alone showed a little effect on lipid level (TC: P > 0.05, LDL: P > 0.05, HDL: P < 0.05). However, compared with HD group, CMS + HD group showed a higher level of LDL (P < 0.01) and a lower HDL lever (P < 0.05) (Figure 3). These statistical analyses testified that high-fat diet exerted a great influence on lipid metabolism, while CMS combined with HD showed a much more influence even though CMS had a little effect on lipid level.

### Effects of high fat diet and chronic stress on liver function

**Figure 4:**
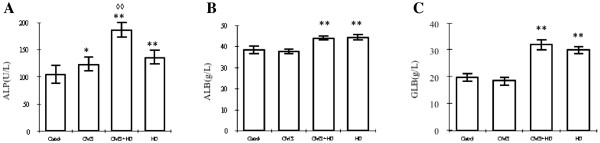
**Effects of stress on hepatic functional enzymes of ALP (A), ALB (B) and GLB(C) in control rats (control), stressed rats (CMS), non-stressed rats fed with HD (HD), and stressed rats fed with HD (CMS + HD)(n = 10 rats per group).** Data were mean ± SEM. ^*^P < 0.05, ^**^P < 0.01: compared to control group; ^◊◊^*P* < 0.01: compared to HD group (one-way ANOVA).

Similarly with influence on lipid metabolism, high fat diet exerted more influence on liver function than chronic stress. Specifically, compared with the concentrations of ALP, ALB and GLB in control group, which were 104 ± 17 U/L, 38.3 ± 1.7 g/L and 19.7 ± 1.5 g/L, respectively, rats had significantly higher levels of ALP, ALB and GLB (All P < 0.01) both in HD group (136 ± 13 U/L, 44.1 ± 1.3 g/L and 29.9 ± 1.3 g/L) and CMS + HD group (186 ± 14 U/L, 43.8 ± 1.0 g/L and 31.9 ± 1.9 g/L). What’s more, rats in CMS + HD group had a higher level of ALP in serum than in HD group, which stated a great statistical difference (*P* < 0.01) (Figure 4).

### HD and CMS showed opposite effects on reverse cholesterol transport (RCT)

**Figure 5:**
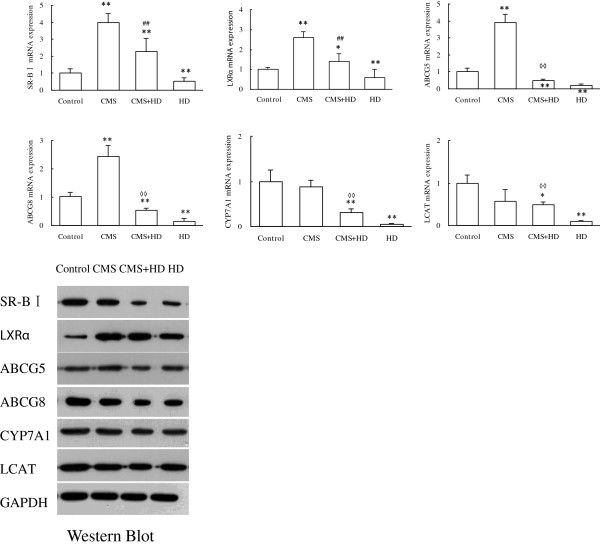
**The results of qPCR showed that high fat diet (HD) significantly decreased the expression of LXRα, ABCG5, ABCG8, SR-BI, CYP7A1 and LCAT,and CMS alone significantly elevated the expression of LXRα, ABCG5, ABCG8, and SR-BI, while CMS + HD significantly decreased the expression of ABCG5, ABCG8, CYP7A1 and LCAT.** The data of mRNA expression were presented as mean ± SEM. ^*^P < 0.05 and ^**^P < 0.01: compared to Control; ^◊◊^*P* < 0.01: compared to HD group; ^##^P < 0.01: compared to CMS group, all with one-way ANOVA. The results of Western blot showed similar trends. The blots shown on gels respectively represented the group of Control, CMS, CMS + HD, HD.

To verify the effects of CMS or/and HD on reverse cholesterol transport (RCT), we examined hepatic mRNA expression of ABCG8, ABCG5, SR-BI, CYP7A1, LXRα, and LCAT using quantitative PCR assays (qPCR). Compared with control group, CMS enhanced mRNA expressions of ABCG8, ABCG5, SR-BI, and LXRα (P < 0.01), and showed little effect on CYP7A1 and LCAT (P > 0.05) while HD reduced all these tested genes’ expression (P < 0.01). We also found that CMS + HD seemed to neutralize the effects of CMS and HD, namely as shown in Figure 5, mRNA levels of all these six genes in CMS + HD group were lower than CMS group but higher than HD group (all the differences are significant), and compared with control, CMS + HD group had up -regulated mRNA expression of SR-BI and LXRα (P < 0.01, P < 0.01) and down -regulated mRNA expression of ABCG8, ABCG5, CYP7A1 and LCAT (P < 0.01, P < 0.01, P < 0.01 and P < 0.05). These results were confirmed by western blotting (Figure 5).

### Histological changes of abdominal aorta and liver

**Figure 6:**
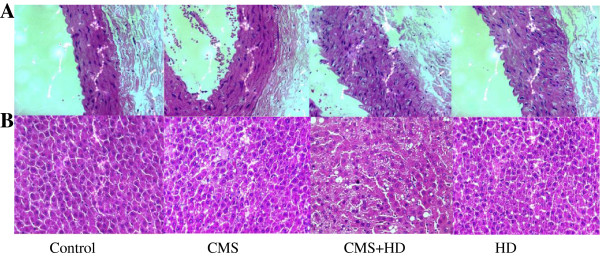
**Photographs of atherosclerotic lesions in the abdominal aorta (A) and liver (B) of rats with HD or/and CMS treatment.** H&E staining was used to analyze the development of atherosclerotic lesions and fatty liver in all groups of control, CMS, HD and CMS + HD. CMS protocol combined with a high-fat diet promoted the formation of unstable aortic plaque and hepatic fatty deposits. Original magnification × 400.

To evaluate structural changes, abdominal aorta and liver sections were stained with H&E. Thickened intima and fibrous layer were observed in HD and CMS + HD group, however, there were no marked histological changes in CMS group, compared to control (Figure 6A). Similarly, severe degeneration associated with fatty deposits was observed, accompanied by inflammatory cell infiltration both in HD and CMS + HD group, and there were no such structural changes in CMS group (Figure 6B).

## Discussion

Although the impact of psychosocial stress on atherosclerosis have frequently been described in clinical and experimental research [20], much less is know about the molecular mechanisms converting psychosocial stress into biochemical changes and protein synthesis dysfunction. In this study, we investigated the relationship between depression-like state and lipid metabolism in rats submitted to the CMS protocol with or without a high-fat diet. The present study shows suffering 2 months of stress, CMS rats and CMS + HD rats both presented depression-like behavior and HD rats and CMS + HD rats both developed atherosclerosis. The effect of stress on lipid metabolism is quite complex. While HD rats had elevated plasma lipid levels, atherogenesis status exacerbated in rats additionally exposed to stress. We found CMS rats had up-regulated expression of hepatic genes related to RCT, which boosted lipid metabolism, while those genes in CMS + HD group were down-regulated. These results are in accordance with a previous study demonstrating that CMS when combined with high-fat diet attenuates lipid metabolism and stimulated atherogenesis. Our observations also implied that CMS alone had no effects on atherogenesis, and reversely it seemed to promote lipid metabolism in the condition of habitual diets. Some studies showed chronic stress altered the blood lipid profile [21,22]. This study suggested the effects of CMS alone were unlikely to be related to plasma lipid abnormalities because stress exposure had little effect on plasma TC and LDL-C.

## Conclusions

In conclusion, our data show that the CMS protocol appears to be an appropriate model for the investigation of the relationship between depression and cardiovascular diseases. The pathogenic effects of CMS with a high-fat diet observed in the study are in agreement with the clinical evidence that depression and vascular diseases commonly coexist. Our study indicated that chronic psychological stress exacerbated atherogenesis not through shifting lipid metabolism, and we proposed the following hypothesis: a constant state of hypervigilance from chronic stress on the basis of HD-induced hyperlipemia, to which animals or humans cannot easily adapt, leads to physical dysregulation and exacerbates atherogenesis. Nevertheless, further studies are needed to explore the allergenic potential of CMS in rats and to explain why CMS induced lipid metabolism disorder only when combined with a high-fat diet.

## Abbreviations

CMS: Chronic unpredictable mild stress; HD: High-fat diet; RCT: Reverse cholesterol transport; TC: Total cholesterol; LDL-C: Low-density lipoprotein cholesterol; HDL-C: High-density lipoprotein cholesterol; ALB: Albumin; GLB: Globulin; ALP: Alkaline phosphatase; LCAT: Lecithin-cholesterol acyltransferase; CYP7A1: Cholesterol 7α-hydroxylase; LXRα: Liver X receptor; SR-BI: Scavenger Receptor BI; ABCG: ATP-binding Cassette transporter.

## Competing interest

The authors report no competing interest. The authors alone are responsible for the content and writing of the paper.

## Authors’ contributions

XLG participated in the design of the study, and performed statistical and genetic analysis and drafted the manuscript. PTL carried out the molecular genetic studies. SQL carried out by the Western blotting. SLW performed blood samples analysis. YEZ conceived of the study, and participated in its design and coordination and helped to draft the manuscript. All authors read and approved the final manuscript.
